# Retraction: Optimization of Precontrol Methods and Analysis of a Dynamic Model for Brucellosis: Model Development and Validation

**DOI:** 10.2196/98667

**Published:** 2026-04-28

**Authors:** JMIR Publications Editorial Office

**Affiliations:** 1 JMIR Publications Toronto, ON

The JMIR Publications Editorial Office is retracting the article:

Optimization of Precontrol Methods and Analysis of a Dynamic Model for Brucellosis: Model Development and Validation [1]

This action is being taken due to identified concerns regarding potential manipulation of the submission process, the integrity of the peer review process, and the relevance of several references.

The concerns with this publication were identified during an investigation of a series of articles with related concerns. In response to our request for comment, author Yihao Huang indicated that the identified references were cited in a broader methodological context and that multiple references may not have been the most appropriate for statements relating to brucellosis transmission or infection. The JMIR Publications Editorial Office determined that the author’s response did not fully resolve the concerns regarding the initial use of these references in the published article.

Yihao Huang disagreed with the decision to retract the article. Author Mingtao Li did not reply directly and could not be reached regarding the decision to retract the article.
